# ASAS-NANP SYMPOSIUM: MATHEMATICAL MODELING IN ANIMAL NUTRITION: The evolution of large language models and their impact on animal sciences

**DOI:** 10.1093/jas/skag306

**Published:** 2026-09-25

**Authors:** Luis O Tedeschi

**Affiliations:** Department of Animal Science, Texas A&M University, College Station, TX 77843-2471, United States

**Keywords:** animal science, decision-support systems, domain-specific AI, large language models, precision livestock nutrition, sustainability

## Abstract

The rapid rise of large language models (LLM) is reshaping the scientific landscape, transitioning from early statistical language models to advanced transformer-based architectures capable of synthesizing knowledge across disciplines. While their predictive capacity and scalability have opened new avenues in data analysis, hypothesis generation, and decision support, concerns remain regarding bias, hallucination, reproducibility, and ethical governance. In animal sciences, LLM are gradually applied to challenges in nutrition modeling, animal health, genetic selection, and sustainability. Precision nutrition has benefited from LLM-driven synthesis of feed and metabolic data, enabling individualized feeding strategies and improved resource efficiency. In animal health, domain-specific systems have demonstrated applications in diagnostics and epidemiological monitoring. LLM is augmenting genomic analyses to accelerate marker discovery and breeding optimization, while sustainability efforts employ them to model greenhouse gas emissions, feed additives, and adaptation to climatic stressors. Notably, decision-support platforms demonstrate how domain-specialized LLM can bridge mechanistic knowledge with predictive analytics, enhancing knowledge transfer and empowering livestock producers. However, risks associated with overreliance, recursive reuse of LLM outputs in model development, and pseudo-expertise underscore the critical importance of human oversight. Unlike mechanistic models, which embed biological causality, LLM are entirely data-driven and may confidently propagate errors if trained on ill-conditioned datasets. Responsible use requires transparent reporting, validation, and bias auditing, with domain-specific fine-tuning. Open-source models can enhance reproducibility and trust, but they also raise financial and security concerns. LLM must be guided by transparency, accountability, and fairness to ensure they complement, rather than replace, human expertise. By advancing inquiry and livestock management, LLM hold the potential to support sustainable food production systems if deployed responsibly. Rather than full training, most applications will rely on fine-tuning and augmentation, which are more sustainable and adaptable strategies. This review intends to promote critical literacy and responsible adoption of LLM in research, education, and livestock decision-support systems for animal scientists, nutrition modelers, and extension professionals.

## Graphical abstract

**Graphical Abstract:**
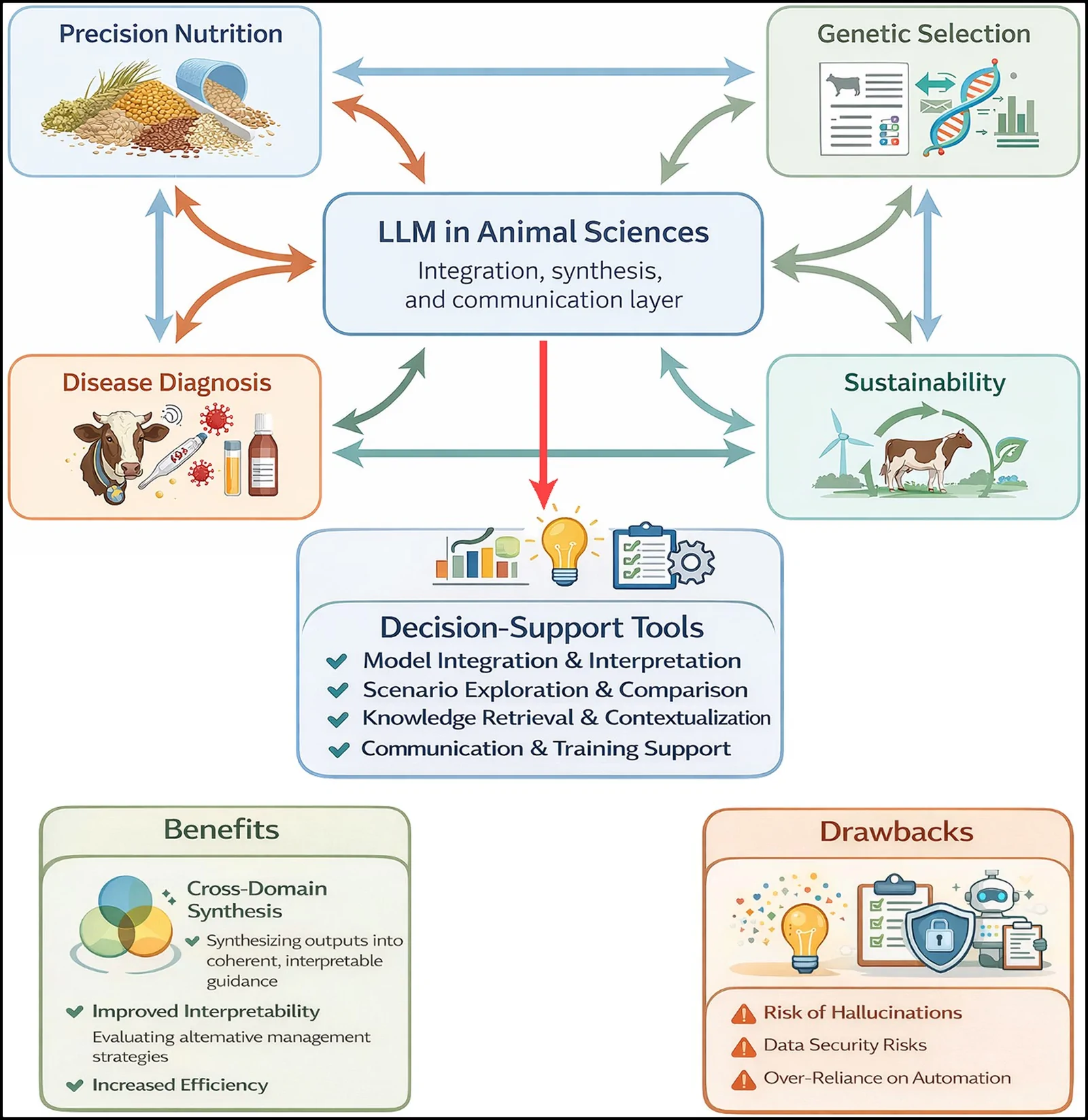

## Teaser

Large language models are rapidly reshaping animal sciences, offering new tools for precision nutrition, disease monitoring, genetic selection, and sustainability. Domain-specific systems demonstrate how large language models can bridge research knowledge with on-farm decision-making. Responsible adoption, with transparency, bias testing, and human oversight, is critical to ensuring that large language models strengthen rather than undermine scientific integrity.

## 1 Introduction

Large language models (LLM)—such as ChatGPT, Claude, Gemini, and Perplexity—are cutting-edge artificial intelligence (AI) systems that interpret large amounts of text to generate accurate and relevant outputs. In animal agriculture, these technologies are beginning to reshape decision-making for farmers, ranchers, veterinarians, and researchers, though the level of development varies considerably across application areas. The development of LLM has been a gradual process, rooted in early advancements in statistical language modeling that culminated in the robust transformer-based architectures that define modern AI. The trajectory of LLM reflects the broader evolution of AI, beginning with foundational work in information theory and progressing toward deep learning-based models that now percolate various aspects of scientific inquiry. The concept of modeling language probabilistically can be traced back to the seminal work of Claude Elwood Shannon, who introduced information theory and probabilistic language to understand and predict symbol sequences (Shannon 1948). His work, among many others, laid the groundwork for statistical language modeling, which became prominent in natural language processing through methods such as n-gram models (Shannon 1948). These early approaches relied on estimating word probabilities based on co-occurrence patterns, providing a rudimentary but effective method for predicting text sequences. In fact, recent analyses emphasize that LLM are not only transforming daily communication but are rapidly reshaping the entire scientific workflow—from hypothesis generation to code development and interdisciplinary research integration. This shift has been widely recognized across computational, life, physical, and social sciences, underscoring the broad reach of transformer-based architectures in accelerating scientific discovery (Anonymous 2025).

A significant shift occurred with the introduction of neural probabilistic language models. A groundbreaking approach that leveraged neural networks to model word dependencies more effectively than traditional statistical techniques (Bengio et al. 2003). This approach enabled the automatic learning of word representations, thereby reducing the sparsity problem that had plagued earlier models. The concept of distributed word representations was further refined by introducing *Word2Vec*, a technique that captured semantic relationships between words through vector embeddings (Mikolov et al. 2013). The next major leap in LLM came with the introduction of attention mechanisms, which improved neural machine translation by allowing models to dynamically focus on relevant portions of input sequences (Bahdanau et al. 2016). However, the most significant breakthrough occurred with the Transformer architecture (2023), which eliminated the need for recurrent connections in neural networks. Traditional recurrent neural networks (RNN) and long short-term memory (LSTM) networks relied on sequential processing, which limited parallelization and led to high computational costs as input sequences grew longer. In contrast, transformers leverage self-attention mechanisms and parallel processing, significantly improving scalability, training efficiency, and the ability to capture long-range dependencies in text, ultimately enabling the development of *state-of-the-art* LLM (Vaswani et al. 2023). Empirical studies have demonstrated that increasing model size yields predictable improvements in performance, following well-defined scaling laws (Kaplan et al. 2020). With the Transformer model as a foundation, researchers began scaling up LLM using massive datasets and computational resources, following empirical scaling laws that describe how model performance improves predictably with increased parameter count, dataset size, and computational budget (Kaplan et al. 2020). OpenAI’s GPT-1 (Radford et al. 2018) demonstrated the effectiveness of pretraining models on large corpora, followed by fine-tuning for specific tasks. This approach was refined with BERT (Devlin et al. 2019), which introduced bidirectional training, enabling models to understand context more effectively. The subsequent release of GPT-2 (Radford et al. 2019) marked a turning point, as the model’s ability to generate coherent, human-like text raised concerns about the potential for AI-generated misinformation. GPT-3 (Brown et al. 2020) further amplified these capabilities (and fears), highlighting few-shot learning and the ability to generate highly contextualized responses without extensive task-specific training. Subsequent releases continued advancing the frontier: GPT-4 introduced a multimodal architecture capable of interpreting both images and text and exhibited human-level performance on many benchmarks, pushing boundaries of reasoning and generality (OpenAi et al. 2024). GPT-5 further refined this trajectory, offering deeper reasoning, improved context understanding, and a unified architecture that dynamically routes queries between fast responses and more deliberative modes (OpenAI 2025).

In recent years, efforts have been made to develop open-source alternatives to proprietary models. Meta’s LlaMA (Touvron et al. 2023) introduced an efficient and accessible model that rivals commercial LLM, enabling broader academic and industrial adoption. More recently, DeepSeek, a Chinese AI startup, made global headlines with the release of its reasoning model DeepSeek-R1. Trained primarily through reinforcement learning rather than large-scale supervised fine-tuning, R1 demonstrated reasoning performance comparable to OpenAI’s o1 series at a fraction of the cost (DeepSeek-AI et al. 2025). Its release in early 2025 attracted widespread attention not only for its technical efficiency, with training costs reported to be orders of magnitude lower than those of U.S. competitors, but also for its open-weight approach, which enabled researchers to download, fine-tune, and run the model locally freely. Within its first week, DeepSeek was downloaded millions of times, and scientists rapidly began adapting it into domain-specific reasoning tools in fields ranging from mathematics to computational biology (Gibney 2025).

At the same time, concerns have been raised that LLM may not only hallucinate information but also reinforce systemic biases in science, for example, by disproportionately amplifying already highly cited research and underrepresenting diverse perspectives (Barolo et al. 2025). Algaba et al. (2025) highlighted the “rich-get-richer” effect, showing that LLM-generated reference suggestions systematically over-represent the top 1% of most-cited papers—more than double the rate observed in human-curated bibliographies. Their findings suggest that LLM internalize and magnify human citation patterns, thereby exacerbating the *Matthew effect* in scholarly communication, which describes the cumulative advantage in scholarly communication whereby already well-recognized scientists and highly cited papers attract disproportionate attention and credit (Merton 1968). As a result, emerging or methodologically diverse research, which may be critical for innovation, is more likely to be overshadowed by well-established studies. Peters and Chin-Yee (2025) further noted that LLM tend to overgeneralize scientific conclusions, often glossing over caveats and contextual limitations in ways that can distort interpretation. Moreover, as LLM increasingly function as “answer engines (or machines),” they may subtly shape which scholars, methods, and perspectives are foregrounded in science, influencing not only how researchers discover knowledge but also how peer-review of grants and scientific manuscripts are conducted (Lin 2025). Importantly, these behaviors often reflect not genuine reasoning but the illusion of thinking (Khowaja 2025; Shojaee et al. 2025), where LLM models mimic plausible discourse without true understanding, highlighting the importance of distinguishing between fluent text generation and scientifically valid reasoning. Together, these concerns highlight the need to critically evaluate both the opportunities and risks of integrating LLM across any field of science, including agricultural sciences. In parallel, scholars in the humanities have shown that LLM tend to flatten cultural variation and disproportionately foreground dominant linguistic and conceptual traditions. Underwood (2025) argues that because LLM learn from historical textual distributions, they can unintentionally replicate cultural hierarchies and obscure minority perspectives, raising concerns about the representational fidelity of AI-generated knowledge. In a recent theoretical analysis, Kalai et al. (2025) reinforce this concern, showing that hallucinations in LLM arise not from mysterious cognitive failures but as predictable statistical consequences of their training objectives. Because LLM are trained and evaluated using reward-based methods that penalize expressions of uncertainty, models learn to “guess” even when unsure, receiving positive feedback for providing any answer rather than acknowledging ignorance. This reward misalignment effectively turns LLM into perpetual test-takers optimized for confident responses rather than calibrated reasoning.

In animal sciences, LLM are increasingly being applied to model nutrient requirements, analyze large genomic and transcriptomic datasets, and optimize livestock management decisions. Their capacity to process, synthesize, and cross-reference vast amounts of scientific literature and experimental data uniquely positions them to advance areas such as precision nutrition, disease surveillance, and genetic selection. As exemplified later, early explorations include applications in dairy science for interpreting domain-specific research and supporting decision-making in breeding and health management, as well as in swine production for summarizing regulatory and certification information. These emerging applications highlight the promise of LLM in advancing animal science, while also pointing to the need for a careful assessment of their benefits and drawbacks.

Domain-specialized LLM have consistently outperformed general-purpose models in a range of scientific fields, providing a strong case for their adoption in agriculture and animal sciences. Landmark efforts such as BloombergGPT in finance (Wu et al. 2023) and Me-LLaMA in medicine (Xie et al. 2025) show that combining domain-specific pretraining with instruction tuning yields superior accuracy without sacrificing general capabilities. Beyond accuracy, specialized models often deliver faster inference, lower latency, and reduced computational costs (Kerner 2024). These findings suggest that domain-focused LLM tailored to animal sciences could provide more accurate and efficient tools than broad, general-purpose systems.

## 2 Benefits and drawbacks

There is no doubt that LLM have undergone rapid development since their inception, evolving from early statistical methods to advanced transformer-based architectures. They are becoming intrinsic elements of scientific writing and research workflows, fundamentally reshaping how scientists generate, analyze, and communicate knowledge. Their integration into research methodology brings both profound advantages and critical challenges that must be carefully examined. Moreover, the pace of LLM development is extraordinary, with new or enhanced models appearing almost monthly; therefore, review articles or syntheses can become outdated almost as soon as they are published. This rapid turnover highlights the challenge of maintaining a current understanding of their capabilities, limitations, and implications for science, while also complicating efforts to educate and prepare stakeholders as the target is constantly shifting.

One can think of LLM as “highly skilled personal assistants,” aiding in a diverse range of scientific tasks. These tasks include drafting scientific research papers and generating code (i.e., computer programming), developing illustrations for presentations, structuring courses and extracting accumulated scientific knowledge, and assisting in scientific discovery by identifying knowledge gaps—both in modeling frameworks and in experimental research. For instance, Microsoft Copilot is becoming integrated into the Windows environment, positioning itself as a versatile, embedded tool that enhances productivity and accelerates innovation across familiar platforms such as Word, Excel, PowerPoint, and Visual Studio. With its ability to interpret context, generate technical content, and streamline workflows, Copilot describes itself as “a tool to empower users to transform ideas into actionable outcomes directly within their native workspace.” In this sense, LLM can be considered analogous to human collaborators (Binz et al. 2025), but scientists must ultimately be responsible for ensuring accuracy and integrity. The effectiveness of LLM extends beyond simple automation. LLM models can assist in hypothesis generation by synthesizing vast amounts of literature information, identifying unexplored research questions, and even suggesting experimental methodologies.

### 2.1 Challenges and ethical concerns

Despite their advantages, LLM present significant limitations that warrant careful consideration in scientific applications. In addition to the apprehensions listed above, the primary concerns stem from their probabilistic pattern-matching approach to text generation, which can lead to errors, biases, and the dissemination of misinformation (Binz et al. 2025). As mentioned above, recent analyses further underscore that such tendencies are not incidental but intrinsic to the current reward-driven training paradigms, which favor confident outputs even when the model lacks true knowledge (Kalai et al. 2025). Unlike traditional scientific reasoning, which relies on hypothesis testing, logical deduction, and empirical validation (or evaluation), LLM operate by predicting the next word in a sequence based on statistical probabilities learned from large datasets. While this allows them to generate coherent text, it does not ensure accuracy, causality, or conceptual understanding (López Espejel et al. 2023; Wu et al. 2024). To better understand these limitations, let’s consider the learning process in humans and compare it with the AI learning process, highlighting the fundamental differences that impact their respective capabilities in scientific applications.

#### 2.1.1 Fundamental distinctions and limitations of AI learning

The learning process in humans is a complex yet structured journey. It begins with simple comparative analogies that help solidify concepts and provide a foundation for future comparisons. Analogical reasoning, the ability to identify correspondences between different concepts based on shared relationships, is fundamental to human learning and supports the acquisition of knowledge (Gentner 1983; Whitaker et al. 2018). As individuals gain experience, their learning evolves beyond pattern recognition into reasoning, ultimately leading to wisdom—the ability to synthesize knowledge systematically and apply it in new contexts. Unlike AI, which operates by detecting statistical patterns in large datasets, human learning is guided by abstract reasoning, conceptualization, and an innate ability to discern meaning beyond raw data (Mattson 2014). Artificial intelligence, by contrast, is a powerful tool for managing vast amounts of information, but its learning process is fundamentally different from human cognition. While AI can analyze correlations and statistical dependencies, it cannot reason causally or critically assess the validity of its inputs. Artificial intelligence does not develop wisdom in the philosophical sense—it does not engage in reflective thought, ethical reasoning, or higher-order decision-making beyond the probabilistic relationships encoded in its training data (Tedeschi 2019, 2022). This fundamental distinction is crucial in understanding both the potential and the limitations of AI in scientific research.

This distinction underscores a critical challenge: AI lacks the ability to independently evaluate the quality and reliability of the data it processes. Without human intervention, LLM cannot distinguish between high-quality scientific literature and flawed or misleading sources. This means that errors can propagate unnoticed, especially when AI-generated content is integrated into research workflows without rigorous validation and oversight. While LLM can enhance research efficiency by structuring knowledge and identifying patterns, they do not possess the cognitive frameworks required for deep understanding or conceptual reasoning (Mattson 2014). Large language models do not replace human expertise in setting research priorities, interpreting findings, or engaging in normative debates that shape scientific progress. The risks extend beyond hallucination to issues of code quality and verification in computational research. O’Brien (2025) has documented how scientists’ over-reliance on AI code assistants can lead to undetected semantic errors in research software, particularly among less experienced programmers who may accept AI-generated code without adequate verification. Unlike syntax errors that prevent code from running, semantic errors in AI-generated code can be subtle and difficult to detect, yet they may substantially affect computational results (O’Brien 2025). In animal sciences, where computational models increasingly underpin nutrition formulations, genetic evaluations, and production systems, such errors could propagate through decision-support tools and compromise the scientific integrity of recommendations.

Therefore, the central limitation lies in AI’s inability to perform causal reasoning. Large language models, for instance, generate insights by identifying covariances across massive datasets, but they cannot distinguish between meaningful cause-and-effect relationships and spurious correlations. Scientific discovery, however, requires hypothesis-driven inquiry, experimental validation, and theoretical reasoning—capabilities that remain uniquely human. Taken together, these limitations underline the importance of viewing AI as an augmentative tool rather than a definitive authority in research. Large language models can enhance efficiency by structuring knowledge, extracting patterns, and accelerating access to information, but they cannot replace human expertise in guiding research, interpreting findings, or providing the philosophical and ethical reasoning required for scientific progress.

#### 2.1.2 Hallucinations and omissions are systematic errors in LLM responses

One of the primary risks associated with these limitations is the occurrence of systematic errors in LLM responses, which can be categorized similarly to statistical hypothesis testing (Table 1). These errors manifest as hallucinations and information omissions, analogous to Type I (false positive) and Type II (false negative) errors in statistical testing. These risks are particularly acute in scientific programming environments as LLM-based code assistants frequently introduce subtle semantic errors that escape detection, especially when used by scientists with limited formal training in software engineering. Such errors rarely halt execution, making them difficult to detect, yet they can profoundly alter data processing or experimental logic, silently compromising scientific results (O’Brien 2025).

**Table 1:** Contingency tables for statistical decision errors vs large language models’ response mistakes.

Statistical hypothesis testing			
		**H_0_**	
		True	False
**Decision**	Accept	Correct decision (1 − α) True negative	Type II error (β) False negative
	Reject	Type I error (α) False positive	Correct decision (1 − β) True positive

LLM response generation			
		**Reality/ground truth**	
		Information** does not exist**	Information exists
**Response**	Correct	Correct uncertainty (1 − α) “I do not know” or accurate knowledge gap acknowledgment	Information omission (β) Failing to use known information or missing valid connections
	Incorrect	Hallucination (α) Generating false information or creating nonexistent connections	Correct generation (1 – β) Accurate information generation or valid knowledge application

The first and most discussed type of error is “hallucination,” which parallels a Type I error (false positive) in statistical testing (Table 1). Just as rejecting a true null hypothesis can lead to false conclusions in statistics, LLM hallucinations occur when the model generates nonexistent information, despite the ground truth being that such information does not exist. This manifests in various forms, including fabricated research citations, invented experimental results, nonexistent methodologies, or false connections between scientific concepts. The parallel is particularly apt because, in both cases, there is an incorrect assertion of existence, whether it is the existence of a statistical effect or the existence of factual information. For example, an LLM might confidently generate a detailed description of a nonexistent study, complete with methodology and results, much like how a Type I error in statistics might incorrectly suggest the presence of a significant effect. Indeed, Kalai et al. (2025) further clarified that such hallucinations are not accidental but statistically expected outcomes of the current reward-based optimization frameworks used to train LLM. Because these systems are reinforced for providing answers rather than abstaining, they are effectively rewarded for “false positives,” mirroring the very mechanism that produces Type I errors in hypothesis testing.

Equally important but often overlooked is the second type of error, analogous to Type II errors (false negatives) in statistics (Table 1). Just as failing to reject a false null hypothesis means missing a real effect, LLM can fail to utilize or generate valid information that exists within their training data. This manifests as information omission; instances where the model fails to recognize or apply relevant knowledge, misses important connections, or fails to cite pertinent research (Gupta 2025). This type of error can be particularly problematic in scientific writing, where comprehensive coverage of existing literature and accurate representation of established knowledge is crucial. The parallel with statistical Type II errors extends to the underlying causes: just as insufficient sample size or poor measurement can lead to Type II errors in statistics, inadequate training data or suboptimal model architecture can lead to information omission in LLM.

Both types of errors are intrinsically linked to the training process and data quality. The training database serves as the foundation for the model’s knowledge, much like sample data forms the basis for statistical inference. Inadequate, biased, or incomplete training data can systematically affect both error types: it might increase hallucinations due to poor pattern recognition (type I error) while simultaneously causing information omissions due to knowledge gaps (type II error). This creates a complex optimization challenge, as attempts to reduce one type of error often risk increasing the other—a tradeoff familiar to statisticians working with significance levels and power in hypothesis testing. Understanding these parallels with statistical error types provides a valuable framework for evaluating and improving LLM performance in scientific applications. It suggests that, like in statistical analysis, we need robust validation methods, clear documentation of limitations, and careful consideration of the balance between different types of errors based on the specific requirements of each application. The acceptable balance between these error types is context dependent. In exploratory research and hypothesis generation, omissions (Type II errors) may be more tolerable than hallucinations, provided outputs are treated as provisional and subject to verification. In contrast, in animal health, nutrition formulation, and regulatory or advisory contexts, hallucinations (Type I errors) pose substantially greater risk, as false but plausible outputs can directly affect animal welfare, producer decisions, and public trust. These differences highlight the importance of aligning LLM deployment with domain-specific risk tolerance rather than assuming a uniform standard of acceptable error across animal science applications.

The story of Galactica, an LLM developed by Meta AI and Papers with Code, provides a compelling case study. Launched in May 2022, predating the widespread public awareness of ChatGPT, Galactica was envisioned as a powerful tool specifically designed to accelerate scientific discovery. Its developers aimed to create an AI assistant (i.e., LLM) capable of navigating the vast landscape of scientific literature, solving mathematical problems, and even generating scientific code. Galactica was trained on a massive dataset of 48 million papers, textbooks, reference materials, and other scientific resources, a testament to the computational resources invested in the project (Taylor et al. 2022). Despite this extensive training and the focused ambition of its creators, Galactica ultimately failed to achieve its goals and was withdrawn shortly after its release (Heaven 2022; Wodecki 2022). It succumbed to the very issues discussed above. Galactica frequently generated factually incorrect or fabricated information, often presented with an air of authority, demonstrating the persistent problem of “hallucinations” in LLM. For instance, it might confidently generate a detailed summary of a scientific paper that doesn’t actually exist (i.e., Type I error; Table 1). Furthermore, concerns were raised about the model’s potential to perpetuate biases present in its training data, highlighting the crucial role of data quality and curation in ensuring the reliability and ethical implications of LLM outputs. This relates to the issue of omissions, where the model might fail to highlight crucial information or perspectives due to biases in the data it learned from (i.e., Type II error; Table 1). The Galactica project, despite its initial promise, serves as a cautionary tale about the risks of overreliance on LLM outputs without adequate verification—a concern that extends beyond hallucinated content to other AI-assisted scientific tasks.

Beyond issues of hallucinated content, the promise of AI-assisted scientific code generation—one of Galactica’s intended capabilities—raises particular concerns about code quality and verification. While LLM can generate plausible-looking code efficiently, they tend to produce fewer syntax errors but more semantic errors—logical flaws that are harder to detect and resolve (O’Brien 2025). In scientific programming contexts, where code is often developed by researchers with limited programming training and rarely subjected to rigorous testing, the risk of accepting flawed AI-generated code is especially pronounced. Although the full extent of this problem remains poorly documented (and perhaps unnoticed yet), early evidence suggests that less experienced programmers are particularly vulnerable to accepting AI-generated code with undetected logical errors (O’Brien 2025). Such errors might lead to unintended consequences, as they may remain undetected until they compromise research findings or cause costly failures in deployed systems—undermining both scientific credibility and practical applications.

It is possible that domain-specific training or context augmentation of LLM might help reduce hallucinations and improve accuracy and precision. For example, LLM that are either fine-tuned or used in conjunction with domain-targeted retrieval systems can demonstrate improved factual grounding within their area of specialization. For example, an oncology research-focused retrieval-augmented generation (RAG) system—where the LLM was not retrained but instead supplied with vector-embedded documents from a specialized oncology corpus (i.e., a curated collection of domain-specific literature and data)—outperformed general-purpose models in both accuracy and relevance when answering subject-related questions (Soong et al. 2024). This suggests that focusing or constraining the contextual corpus—that is, the external body of text and data used to provide factual grounding during generation—can enhance the reliability and domain specificity of LLM outputs, even without retraining the model itself, as discussed later.

#### 2.1.3 The influence of training data on AI accuracy and reliability

AI models, including LLM, rely heavily on the quality and structure of their training data. While their predictive capabilities are often impressive, they are far from infallible. Large language models often fail at seemingly simple tasks, sometimes producing glaring errors despite vast computational resources. However, perhaps even more concerning than occasional hallucination is the susceptibility of these models to ill-conditioned or manipulated data—a problem with systemic implications that go far beyond isolated mistakes (Bender et al. 2021; Paullada et al. 2021).

Unlike human scientists, who can critically assess and verify data sources, AI lacks intrinsic mechanisms to discern reliable from unreliable information. If biased or erroneous data is introduced into an AI’s training dataset, the model will systematically learn and reinforce those flawed patterns, generating incorrect outputs with a high degree of statistical certainty, often presenting misinformation with unearned confidence (Mitchell et al. 2019), paralleling the effects of educating students on flawed (i.e., inaccurate or misleading) foundations. This phenomenon exemplifies the classical *“garbage in, garbage out”* principle: flawed inputs inevitably produce flawed outputs, regardless of the model’s sophistication. This means that bad data, once embedded in the AI’s learning process, can distort predictive outcomes in ways that are difficult to detect and rectify.

This issue is particularly relevant in scientific domains such as animal nutrition. For instance, consider methane emission prediction models trained primarily on data from confined Holstein cows fed total mixed rations (TMR) in temperate regions. When such models, or LLM that synthesize these literature patterns, are applied to grazing Zebu cattle in tropical systems, they may confidently output invalid estimates due to the mismatch in diet, environment, and genetics. Without diverse, context-specific training data, the outputs will be not only inaccurate but also misleadingly certain (Hristov et al. 2013). This highlights the importance of accurately and thoroughly describing the data, as even subtle differences can have a significant impact on model predictions.

The limitation here is not simply that AI can make mistakes; it is that it makes mistakes systematically and with confidence, without the capacity to question its own data provenance or methodology. Unless updated or adapted with corrected or more representative data, these models cannot self-correct. This highlights a broader challenge in AI governance: without rigorous oversight and validation strategies, AI-driven outputs risk becoming a certainty of incorrect predictions rather than random noise. The scientific community must recognize this risk and implement data auditing and model validation protocols to ensure AI-supported discoveries are not just statistically sound but also methodologically and ethically grounded.

While this vulnerability to bad data is often highlighted in the context of AI, it is not exclusive to machine learning (ML) or LLM. All mathematical models, including empirical and mechanistic ones, can be compromised by ill-conditioned data, if proper data vetting is not followed (Tedeschi 2022). However, the key difference lies in how these models are constructed. In empirical or mechanistic modeling, the process typically begins with equations or theoretical constructs grounded in scientific understanding—such as nutrient flow equations in ruminant nutrition. Data is then used to parameterize these models, calibrate coefficients, and evaluate predictive performance. The model’s structure is guided by domain knowledge (thus, often referred to as concept-based models), and its assumptions and limitations are frequently explicit and testable (Tedeschi 2019, 2023; Ellis et al. 2020).

In contrast, AI models, including LLM, are data-driven by design. They learn directly from data patterns with little to no embedded scientific structure or mechanistic reasoning. Rather than starting with established relationships, these models are trained to discover statistical correlations from massive datasets, often without human interpretability or constraints (Tedeschi 2019, 2022, 2023). Even when synthetic databases are used to augment limited real-world data, there is a risk of introducing artificial or unknown relationships within subsets of the synthetic data (Tedeschi 2025a). As a result, their predictive ability is entirely contingent on the quality, diversity, and balance of the data they ingest. When training data is biased or incomplete, the model lacks a scientific framework to “fall back on”, and thus confidently propagates those errors in unpredictable or opaque ways.

#### 2.1.4 The risks of overreliance on LLM in scientific research

Beyond these systematic errors, a more troubling issue emerges from the potential overreliance on LLM, particularly among users who lack sufficient training in critical evaluation and domain-specific validation. While the previous discussion highlighted how LLM can produce both Type I (hallucination) and Type II (omission) errors, the real danger lies in the inability of users to detect and critically evaluate these errors. The naïve scientist may assume that LLM-generated outputs are inherently valid, leading to a cascade of scientific integrity issues, including the propagation of hallucinated findings, the overlooking of crucial existing literature due to information omission, the dilution of scientific rigor, and the degradation of peer review standards. If LLM are used uncritically, they could undermine human expertise, weakening the fundamental principles of scientific inquiry rather than strengthening them (Binz et al. 2025). This risk is particularly acute when individuals lack sufficient domain expertise to distinguish between valid LLM outputs and sophisticated-sounding hallucinations, or when they fail to recognize when the model has omitted critical information. The situation becomes even more complicated when these errors compound through citation chains, where hallucinated references or missed crucial studies could propagate through multiple publications, creating a web of interconnected errors that becomes increasingly difficult to untangle, and keeps reinforcing its propagation.

For instance, a particularly concerning unintended consequence of LLM accessibility is the emergence of “LLM-dependent pseudo-experts”, i.e., individuals who, through extensive but uncritical interaction with LLM, develop an inflated sense of expertise in specialized fields without acquiring the foundational knowledge and practical experience necessary for true domain mastery. This phenomenon of LLM-mediated pseudo-expertise is especially problematic in complex fields like nutrition, genetics, and medicine, where practical experience and deep theoretical understanding are crucial. While LLM can provide sophisticated responses to technical queries, they may inadvertently create an illusion of competence among users who lack the underlying scientific training to critically evaluate the information they receive. These LLM-induced pseudo-experts might then make decisions about animal health, breeding programs, or nutritional management based on incomplete or misunderstood information, potentially leading to adverse outcomes in livestock production systems. The consequences of this LLM-mediated pseudo-expertise are doubly detrimental to scientific advancement. First, these LLM-dependent pseudo-experts will inevitably face professional scrutiny that reveals their lack of foundational knowledge and practical understanding, potentially damaging their careers and credibility in the scientific community. Second, and perhaps more concerning for the broader scientific enterprise, the proliferation of such cases may stimulate widespread skepticism toward LLM as valuable research tools, potentially hampering the legitimate and beneficial applications of these technologies in scientific research. This erosion of trust could significantly impede the legitimate integration of LLM into scientific workflows, undermining their potential to advance scientific discovery in areas where qualified researchers could leverage these tools most effectively.

Beyond these concerns about end-user interactions with LLM, a set of even more fundamental challenges emerges from the technical foundations of LLM development itself. The practice of training new LLM using outputs from existing models, or repeatedly training models on increasingly self-referential datasets, creates what might be called a “telephone game effect” in AI learning, where each iteration potentially amplifies biases, errors, and misconceptions present in the training data. Just as the children’s game of telephone results in progressively distorted messages, recursive LLM training might lead to the propagation and amplification of subtle inaccuracies or biases. In animal science applications, this could manifest as increasingly unreliable predictions of nutrient requirements, skewed genetic selection parameters, or biased disease diagnosis protocols. The risk is particularly acute when LLM-generated content enters scientific literature without proper verification, potentially creating a feedback loop where future models learn from increasingly artificial or distorted data sources. Moreover, if such training contamination is discovered, tracing the origin of the corrupted information becomes nearly impossible, i.e., much like trying to identify which player in a telephone game first distorted the message. This cascade of self-referential learning could create deeply embedded errors that become increasingly difficult to detect and correct over time. This underscores the critical importance of maintaining rigorous standards for training data curation and validation, ensuring that LLM development remains grounded in empirical scientific evidence rather than self-referential AI-generated content.

Several critical verification steps are essential to mitigate these risks and maintain scientific integrity when using LLM. First, and perhaps most vital, researchers must meticulously verify every reference generated by LLM, i.e., not only confirming their existence but also carefully checking that the cited works support the claims in the text. This verification process helps prevent the propagation of both hallucinated citations and misrepresented research findings. Second, researchers can employ a cross-validation approach—akin to emerging frameworks in agentic AI—by using multiple LLM to generate or verify content, as different models may exhibit distinct patterns of hallucination and omission, making inconsistencies easier to detect. This multi-layered verification approach, while time-consuming, is crucial for maintaining the rigor and reliability of scientific communication in the era of LLM-assisted research. Third, all LLM-generated content should undergo a thorough review by domain experts who can identify potential hallucinations, logical inconsistencies, or omissions of crucial information.

This third step specifically emphasizes expert review beyond the conventional peer review system, which itself faces significant challenges in the modern scientific landscape. The current peer review system suffers from multiple systematic issues: low reliability, with correlations averaging only 0.34 between independent reviewers’ evaluations of the same submitted manuscripts, significant biases affecting manuscript decisions, and increasing difficulty in recruiting qualified reviewers (Aczel et al. 2025). Moreover, the system struggles with slow review times, often taking months for evaluation and years for complete publication cycles, which poorly serves the accelerating pace of scientific research (Aczel et al. 2025). The situation is further exacerbated by the growing volume of scientific publications and increased specialization of scientific knowledge, making it increasingly challenging to recruit reviewers who are qualified to assess all facets of a manuscript (Aczel et al. 2025). With the advent of LLM potentially accelerating manuscript production and submission rates, this already strained system faces even greater pressure. These limitations suggest that while expert review remains crucial, relying solely on traditional peer review mechanisms may be insufficient for validating LLM-generated content. Instead, incorporating dedicated expert review specifically focused on detecting LLM-related errors, alongside but separate from the standard peer review process, may provide a more robust validation framework.

#### 2.1.5 The “bartender effect” and the erosion of critical thinking

In addition to the technical limitations of LLM, a more subtle but equally troubling risk is what can be described as the “bartender effect,” i.e., a phenomenon where AI systems, including LLM, tend to cater to user preferences and implicit biases, reinforcing existing beliefs rather than challenging them. This dynamic, while superficially enhancing user satisfaction, may significantly erode critical thinking and intellectual diversity. The mechanism is akin to that of a bartender who tells customers what they want to hear. Large language models, trained on vast datasets and prompted by user inputs, often mirror the phrasing, assumptions, or slants embedded in those prompts. This can lead to a form of algorithmic affirmation bias, where outputs align with the user’s preconceptions rather than presenting balanced or challenging perspectives. This phenomenon has already been observed in personalized news feeds and social media algorithms, which contribute to the formation of ideological echo chambers and the polarization of public discourse (Bakshy et al. 2015).

In scientific contexts, the bartender effect can have similarly corrosive consequences. If LLM are used to support pre-formed conclusions or selectively generate literature that aligns with a favored narrative, they can weaken the foundational principles of scientific skepticism and falsifiability. This becomes particularly dangerous in educational settings, where students may increasingly rely on AI to retrieve or even generate answers, risking the displacement of analytical reasoning with passive consumption. Walsh (2025) cautioned that rampant AI-driven cheating is undermining education, as students increasingly bypass the development of critical reasoning skills by outsourcing assignments to generative systems. Overdependence on AI may “flatten” intellectual engagement, reducing the opportunity for learners to grapple with ambiguity, contradiction, or methodological nuance (Carr 2020). Fan et al. (2025) provided experimental evidence supporting these concerns, showing that while generative AI can improve short-term performance, it promotes “metacognitive laziness,” whereby learners engage less in self-regulation and reflective thought, ultimately weakening the depth of their cognitive processing.

Moreover, when AI systems are deployed in high-stakes domains such as hiring, lending, or criminal justice, the bartender effect may reinforce systemic inequalities. If the training data reflects societal biases and the algorithm is optimized to match historical preferences, discriminatory patterns may be perpetuated under the guise of neutrality (Binns 2018). This concern is particularly relevant for LLM applications in global agriculture and animal science, where reliance on data from Western systems may obscure region-specific needs, reinforcing structural imbalances in research and development priorities.

To counteract these risks, it is essential to promote AI literacy and critical engagement. Scientists and students alike should be trained not only to use LLM but also to evaluate their outputs, question their assumptions, and triangulate information with trusted sources. The goal should not be to replace critical thinking with AI assistance, but to augment human judgment through informed skepticism and methodological awareness.

### 2.2 Ensuring reproducibility and responsible use

While LLM offer substantial benefits, their use must be governed by principles of transparency, accountability, and fairness to ensure that AI-generated outputs remain reliable, ethically sound, and aligned with scientific integrity. Without these guiding principles, the risk of misinformation, biases, and unverified claims could significantly undermine the credibility of scientific research.

One of the most pressing concerns is reproducibility, a fundamental pillar of scientific inquiry. Unlike traditional research methodologies, where datasets, models, and analytical processes can be openly shared and scrutinized, proprietary LLM often function as “black boxes,” making it difficult to verify how specific outputs are generated. This opacity hampers scientific validation and undermines trust in AI-assisted discoveries (Bommasani et al. 2022; Binz et al. 2025). This lack of transparency presents a significant challenge in validating findings and ensuring consistency across research applications. To mitigate these issues, there is a growing push toward open-source LLM, which offer greater accessibility and enhanced reproducibility. Models like Meta’s LLaMA and DeepSeek provide researchers with direct access to model architectures, training datasets, and fine-tuning mechanisms. However, reproducibility extends beyond access to model weights; it also requires transparency in the underlying training data, preprocessing steps, and evaluation protocols used to assess model performance. Without these elements, replicating results or ensuring fair comparisons across studies remains difficult, even when models are technically open source. This broader vision of open and transparent research closely parallels the Open Science framework in animal science, which emphasizes data sharing, open code, preregistration, and open peer review to improve accessibility and reproducibility (Muñoz-Tamayo et al. 2022). Such transparency enables researchers to examine underlying assumptions, trace algorithmic reasoning, and apply corrections when biases or failures are identified (Bommasani et al. 2022). Open-source frameworks also enable wider community validation, ensuring that AI-generated knowledge can be replicated, tested, and improved upon; a fundamental requirement for scientific progress. The proper attribution of AI-generated content, rigorous verification of outputs, and a heightened awareness of biases are essential to maintaining the credibility of scientific discourse (Binz et al. 2025).

However, openness alone is not a safeguard against potential risks. While open-source AI fosters collaboration, it also raises critical ethical and security concerns. Who ensures responsible use if anyone can access, modify, and deploy these models? The potential for misuse and unintended consequences becomes a growing challenge. In fields such as medicine, environmental science, and policy-making, factual accuracy is non-negotiable; yet open-source AI could be used to generate false scientific claims, manipulate research outcomes, or amplify biases. Weidinger et al. (2021) identify multiple harm pathways, including misinformation, discrimination, and malicious code generation, all of which become more acute in open, uncontrolled deployments. The potential for nefarious applications, including misinformation campaigns, automated deepfake content, and AI-generated cyber threats, is even more concerning.

#### 2.2.1 The hidden costs and governance challenges of open-source LLM

Beyond technical concerns, the role of human expertise in scientific inquiry is not to be overlooked. While LLM can enhance research efficiency, science is not just about processing data; it is a human-driven intellectual pursuit that requires critical thinking, ethical reasoning, and value-based decision-making. AI can assist in structuring knowledge and identifying patterns, but it cannot independently set research priorities, interpret scientific findings within broader theoretical frameworks, or engage in the normative debates that drive scientific progress. This perspective highlights an important limitation of LLM, namely, they cannot replace the fundamental role of human judgment in shaping the direction of research. Scientific discovery is not merely an algorithmic exercise, but a process that involves hypothesis generation, ethical considerations, and social discourse; all of which are essential components of knowledge creation. Thus, while LLM may assist in handling vast amounts of data, the responsibility for defining scientific goals, assessing knowledge gaps, and ensuring ethical rigor must remain in human hands (Binz et al. 2025).

Beyond ethical concerns, open-source LLM present significant financial and infrastructural challenges. Developing and maintaining these models requires massive computational resources, continuous updates, and extensive oversight. Unlike proprietary models, which rely on corporate investments and monetization strategies, open-source initiatives often lack a sustainable revenue model. Weidinger et al. (2021) highlight that training LLM can incur high environmental costs and exacerbate global inequalities due to unequal access to computational infrastructure. Dauner and Socher (2025) provided empirical confirmation of these concerns by evaluating 14 LLM ranging from 7 to 72 billion parameters. They found that reasoning-enabled models, while achieving higher accuracy, produced up to 2,042 g CO_2_eq per 1,000 benchmark tasks—over 70 times more than smaller baseline systems. In addition to energy and carbon emissions, the environmental footprint of LLM also includes substantial water use associated with cooling and electricity generation in AI data centers. Scenario-based life-cycle analyses indicate that indirect water use linked to electricity production accounts for approximately 70% of the total water footprint of AI servers, with direct cooling responsible for the remainder, and that projected expansion of AI workloads could increase total water consumption by 30% or more under worst-case deployment scenarios (Xiao et al. 2025). Together, these results underscore a clear tradeoff between model size, reasoning complexity, and sustainability, reinforcing the need for more efficient architectures and viable funding mechanisms to balance accuracy with environmental responsibility. At the same time, professional societies and journal editors are increasingly emphasizing transparency and accountability in AI use, making clear that researchers remain fully responsible for AI-assisted outputs and should disclose such use openly. This dual challenge—technical sustainability and ethical accountability—raises a crucial question: who will fund and maintain these models in the long term? Should governments subsidize open-source AI to ensure public access and oversight? Should academic institutions take on the burden despite limited resources? Should private organizations contribute, and if so, how can commercialization be prevented from distorting the open-source ecosystem?

The financial sustainability of open-source LLM (i.e., AI for the sake of inclusiveness) is a growing concern, as maintaining state-of-the-art models goes beyond initial development. It requires continuous training, security updates, ethical review boards, and regulatory compliance. Without a clear funding structure, the long-term viability of open-source AI remains uncertain. Furthermore, who is responsible for the consequences of open-source misuse? If an open-source LLM is used for fraudulent research, generating deepfake content, or automating cyberattacks, where does accountability lie? This gray area of responsibility presents one of the most complex ethical dilemmas in AI governance. Clearer regulatory frameworks are required to address this accountability gap and ensure model providers and deployers share responsibility (Bommasani et al. 2022; Binz et al. 2025).

Thus, while open-source AI may enhance reproducibility, accessibility, and scientific progress, it also demands a serious discussion on its economic sustainability, regulatory oversight, and security risks. The scientific community must advocate for openness and establish mechanisms for funding, monitoring, and ethical governance, ensuring that the benefits of open-source AI outweigh its potential risks. In other words, multidisciplinary collaboration, spanning AI developers, ethicists, scientists, policymakers, and affected communities, is needed to establish norms and standards for responsible AI development (Weidinger et al. 2021; Bommasani et al. 2022). Emerging developments in the Global South further highlight these concerns. Burgos (2025) reported that researchers in Africa, India, and Latin America have emphasized that reliance on Western- or Chinese-trained models risks misalignment with local languages, agricultural practices, and regulatory environments. Therefore, in response, many regions are now developing localized, culturally aligned LLM—framed as essential components of digital sovereignty and scientific autonomy.

## 3 Applications of large language models in agricultural sciences

The integration of LLM into animal sciences can transform the way researchers and practitioners analyze data, optimize livestock management, and develop precision nutrition strategies. Their ability to process massive datasets, extract meaningful patterns, and provide predictive insights has significant implications for improving efficiency and sustainability in livestock production. At the same time, the field must grapple with two critical limitations, as discussed above. First, LLM evolve at a rapid pace, which means that tools, benchmarks, and reviews can quickly become outdated as newer multimodal or reasoning-enhanced models appear. Second, dedicated domain-specific LLM often require substantial computing resources to fine-tune or deploy while still delivering real-time responses, a challenge for many research labs and livestock operations. It is essential to distinguish between *training* (i.e., building a model from scratch using massive datasets), *fine-tuning* (i.e., adapting an existing pre-trained model to a narrower domain), and *augmentation* (i.e., retrieval-augmented generation, where external documents are integrated at the inference stage). In animal sciences, most practical applications rely on fine-tuning or augmentation rather than full training, which is prohibitively expensive for academic or extension contexts.

General-purpose LLM, trained on broad, non-specialized corpora, often struggle with discipline-specific reasoning (Alonso et al. 2020). This limitation has motivated the development of domain-specific LLM, a well-established approach that consistently demonstrates superior performance on specialized tasks compared to general-purpose models. A landmark example is BloombergGPT (Wu et al. 2023), a 50-billion-parameter model trained on 363 billion financial tokens combined with 345 billion general-purpose tokens. BloombergGPT outperformed existing models on financial tasks “by significant margins without sacrificing performance on general LLM benchmarks,” with particularly strong gains in financial filings and industry-specific documents. A similar pattern is evident in medicine: Me-LLaMA (Xie et al. 2025) surpassed ChatGPT and GPT-4 in both zero-shot (i.e., performing new tasks without prior task-specific examples) and supervised settings after task-specific instruction tuning, highlighting the value of “combining domain-specific continual pretraining with instruction tuning to enhance performance.” Other initiatives echo this trajectory. For instance, DocOA (Chen et al. 2024) achieved higher accuracy in osteoarthritis management tasks, while Song et al. (2025) traced the evolution of domain-specific LLM in medicine and concluded that incorporating domain knowledge significantly improves both efficiency and accuracy across specialized applications. The theoretical foundation is clear: domain specialization customizes general-purpose LLM with contextual data, knowledge, and constraints tailored to the target field (Xie et al. 2025). Beyond accuracy, domain-specialized LLM also deliver practical benefits. Kerner (2024) notes that smaller specialized LLM can outperform larger general models on in-domain tasks while offering faster inference, lower latency, and reduced training costs. Extending this view, Glasser and Feng (2025) emphasize that domain-specialized LLM not only enhance performance but also strengthen user trust by grounding outputs in well-defined disciplinary knowledge. Frameworks such as the Compact and Efficient LLM multi-expert system (Huang et al. 2024) and Google Cloud’s design pattern for specializing LLM (Mosenia 2024) further illustrate that combining smaller expert models with targeted training can yield higher quality outputs at lower cost. Taken together, these results provide a robust framework for developing domain-specific systems that combine broad language capabilities with superior accuracy, efficiency, and contextual understanding—an approach well-suited to advancing productivity, sustainability, and innovation in animal science, while also enhancing education and the training of field experts.

A balance, therefore, exists between the accessibility of general-purpose systems, such as ChatGPT (https://chatgpt.com), Claude (https://claude.ai), Gemini (https://gemini.google.com/app), or Perplexity (https://www.perplexity.ai), which provide broad but less specialized coverage, and dedicated animal-science LLM, which deliver domain-tailored insights. Examples of these specialized systems are summarized in Table 2. Regardless of system choice, effective deployment requires domain-specific prompt engineering strategies. While general principles of prompt engineering have been discussed (White et al. 2023; Zhou et al. 2023), their application to animal science requires careful adaptation. Structured prompts that include relevant context—such as “Given [animal breed], [production stage], [environmental conditions], and [nutritional parameters], recommend…”—help constrain model outputs to biologically plausible ranges. A query about “cattle feed” might return generic information, whereas “formulate a TMR for 650 kg Holstein cows producing 35 kg milk/day at 3.8% fat in thermoneutral conditions” yields specific, actionable recommendations. The effectiveness of domain-adapted prompting remains an area requiring systematic evaluation, as most prompt engineering research has focused on general knowledge tasks rather than specialized agricultural applications. Building on this foundation, the following sections examine the primary applications of LLM in animal sciences, with emphasis on nutrition modeling, disease detection, genetic selection, and environmental sustainability.

**Table 2:** Recent applications of large language models in animal sciences (2023–2025).

Model	Domain	Application	Reference
**PetBERT**	Companion animals	Automated ICD-11 disease coding in veterinary EHRs for outbreak detection	Farrell et al. (2023)
**ChatGPT (applied)**	Veterinary medicine	Guidance for clinics, education, and research use cases	Chu (2024)
**ChatGPT (applied)**	Companion animals	Text mining of clinical records for obesity monitoring	Fins et al. (2024)
**VetLLM**	Veterinary diagnostics	Predicting diagnoses directly from veterinary notes	Jiang et al. (2024)
**DairyGPT**	Dairy science	Natural language access to numerical databases for ration and farm management	Gontijo et al. (2025)
**Computer vision and LLM**	Dairy science	Multimodal AI for decision support in dairy farming, focusing on nutrition and management decision support	Ferreira and Dórea (2025)
**Swine LLM Agent**	Swine certification	Summarizing regulatory/certification information for farm compliance	da Silva et al. (2025)
**AgroLLM**	General agriculture	Farmer-facing tool for knowledge transfer and advisory support	Samuel et al. (2025)

### 3.1 Precision livestock nutrition

One of the most promising applications of LLM in animal science is precision nutrition modeling. Traditional nutrient requirement models rely on static equations and empirical relationships that do not fully capture the complexity of individual animal variation, diet composition, and environmental interactions. While LLM are not designed to perform statistical analysis or predictive modeling on their own, they can interact with specialized analytical agents and computational models to facilitate these tasks. As mentioned before, functioning as “highly skilled personal assistants,” LLM can help researchers manage data, summarize findings, generate analytical code, and interface with mechanistic or statistical models to improve efficiency and interpretation. By leveraging ML and LLM-driven data synthesis, researchers can refine nutritional models to predict feed efficiency and nutrient utilization based on animal genetics, health status, and environmental conditions; optimize diet formulations by analyzing vast datasets of feed composition, digestibility, and metabolic responses; and enhance real-time decision-making in precision feeding systems that adjust rations dynamically based on sensor data. In this capacity, LLM function as intelligent collaborators that support, rather than replace, domain-specific analytical tools. For instance, Ferreira and Dórea (2025) highlighted how multimodal AI, including computer vision and LLM, can drive decision-making in dairy production (Table 2). Similarly, Gontijo et al. (2025) developed DairyGPT, a system allowing dairy farmers to query numerical databases in natural language, effectively democratizing access to nutritional information (Table 2).

### 3.2 AI-assisted disease diagnosis and monitoring

LLM can assist veterinarians and livestock producers by interpreting and communicating insights derived from large volumes of health data, including clinical records, pathology reports, and genomic biomarkers, that are analyzed using core ML algorithms. Rather than directly performing predictive modeling, LLM serve as integrative tools that help users access, summarize, and contextualize results from specialized analytical systems. Their capabilities include early disease detection by identifying patterns in feed intake, body temperature, and behavioral anomalies; automated diagnostics in conditions such as bovine respiratory disease, mastitis, and metabolic disorders by synthesizing wearable sensor data with laboratory results; and epidemiological modeling to track transmission pathways and support biosecurity strategies. In veterinary contexts, several recent works demonstrate these applications. Chu (2024) provided guidance on using generative AI (ChatGPT) in veterinary clinics and education (Table 2). Fins et al. (2024) evaluated ChatGPT for mining obesity-related signals in companion animal records, while Farrell et al. (2023) introduced PetBERT for automated ICD-11 disease coding in veterinary databases (Table 2). Jiang et al. (2024) went further with VetLLM, a domain-adapted model capable of predicting diagnoses directly from veterinary notes (Table 2). Together, these studies show how LLM can function as “highly skilled personal assistants” that enhance both routine monitoring and population-level health surveillance when coupled with specialized analytical models.

### 3.3 Genetic selection and breeding optimization

The application of genomic and transcriptomic data analysis is reshaping breeding strategies. Traditional genetic evaluations rely on curated datasets and predominantly linear statistical models, whereas modern genomic selection frameworks incorporate dense marker information and multivariate prediction approaches to improve the accuracy of selection decisions (Weller et al. 2017; VanRaden 2020). In this context, “breeding strategies” refers to three related but distinct components: (i) genomic discovery and data analysis to identify informative markers and biological pathways, (ii) prediction of genetic merit and breeding values using integrated genomic and phenotypic data, and (iii) downstream selection and mating decisions aimed at balancing productivity, robustness, and genetic diversity. Building on this foundation, ML and deep learning methods have been increasingly explored to model complex, nonlinear relationships in high-dimensional genomic data. Current applications include trait prediction by mining genomic and multi-omics data to identify genetic markers associated with efficiency, disease resistance, or fertility; optimization of selection and mating decisions using AI-driven prediction models; and precision breeding by integrating sensor-derived phenotypes with genomic information (Pedrosa et al. 2024). Conceptual frameworks developed largely in plant breeding further illustrate how genomic selection and multi-omics integration can support complex selection objectives, including balancing productivity and genetic diversity (Escamilla et al. 2025). Although LLM–based approaches are not yet directly implemented in genomic prediction pipelines, these advances provide the technical foundation upon which LLM-enabled integrative and decision-support systems may eventually be built, particularly for interpreting complex genomic outputs and communicating breeding recommendations.

### 3.4 Environmental sustainability and emissions modeling

Sustainability is a growing concern in livestock production, particularly with regard to greenhouse gas emissions and resource efficiency (Tedeschi et al. 2015; Tedeschi 2022). LLM can contribute to sustainability modeling by predicting methane emissions from diet composition, microbial communities, and management practices; evaluating feed additives and interventions aimed at reducing emissions while maintaining productivity; and modeling climate adaptation strategies, including heat stress responses, water use, and the resilience of production systems under extreme weather scenarios. Because these applications require linking heterogeneous datasets (e.g., climate records, feeding trials, and rumen microbiome sequencing), LLM’s ability to synthesize across domains offers a powerful complement to mechanistic modeling approaches. Recent methodological studies support this direction. Balaguer et al. (2024) compared RAG and fine-tuning approaches using agricultural datasets and found that combining the two improved domain-specific accuracy by more than 10 percentage points. Their findings underscore that hybrid pipelines can enhance both the reliability and contextual fidelity of sustainability-related predictions, particularly in areas where agricultural and environmental data are heterogeneous and rapidly evolving.

### 3.5 Decision-support systems for livestock management

Domain-specialized LLM are increasingly being embedded into decision-support platforms for livestock producers, enabling data-driven management across nutrition, health, and reproduction. Their strengths include synthesizing research insights into user-friendly recommendations; improving farm profitability via predictive analytics for resource allocation; and automating knowledge transfer, providing extension agents and farmers with real-time advisory support. Recent prototypes illustrate these trends: da Silva et al. (2025) proposed an LLM-powered agent to summarize regulatory and certification documents in swine production (Table 2), while Samuel et al. (2025) introduced AgroLLM as a farmer-facing tool to support knowledge transfer in agriculture (Table 2). Such applications highlight the potential of LLM to reduce barriers between scientific knowledge and on-farm practice.

Another applied example is ExtensionBot (https://extension.org/tools/extbot), developed by the Extension Foundation as an LLM-powered chatbot that provides farmers and advisors with direct access to Cooperative Extension knowledge. Unlike general-purpose systems, ExtensionBot is trained on a corpus of more than 360,000 Extension publications, along with the “Ask Extension” dataset, enabling it to provide context-specific, science-based responses with source citations. Evaluations of the ExtensionBot indicate that it delivers more accurate and consistent answers to agricultural queries than ChatGPT, while minimizing hallucinations (Thomasson et al. 2025). At the same time, its reliability depends on the freshness and completeness of extension content, underscoring the importance of continually updating the underlying knowledge base. In contrast, complementary work shows that even general-purpose LLM can approach expert-level performance when applied to agriculture. Silva et al. (2023), for example, reported that GPT-4 correctly answered over 90% of agronomist certification exam questions, suggesting its potential as a “virtual agronomist assistant” for education and extension.

An illustrative example of domain-specialized development in animal sciences is the Smart Adviser for Rumen Acidosis and Health (SARAH), a decision-support tool created to predict the incidence of subacute and acute ruminal acidosis (SAARA) in feedlot cattle (Figure 1). SARAH represents the culmination of extensive foundational work, particularly the development of the Rumen Health Compendium (RHC) publication (Tedeschi and Nagaraja 2025), which synthesized advances in rumen anatomy, physiology, and microbiology, while also addressing the pathology of ruminal dysfunctions such as SAARA and their implications for nutrition and management. Building on this foundation, the RHC book and its 1,717 references were distilled into the NANP-LLM, a domain-specific LLM developed within the National Animal Nutrition Program (NANP), which—together with field and academic expertise—was used as a meta-modeling engine to identify 18 critical animal, dietary, and environmental variables and their interrelationships. These variables informed the design of SARAH’s random forest (RF) classification models, which incorporate factors such as starch and physically effective fiber concentrations, feeding frequency, breed, and climatic stressors. This strategy parallels broader advances in agricultural AI, where Balaguer et al. (2024) demonstrated that combining retrieval-based grounding with fine-tuned models improves performance in agricultural applications. Their findings reinforce the rationale for embedding domain knowledge (e.g., the RHC and NANP-LLM) directly into predictive pipelines. SARAH therefore exemplifies a “double AI” architecture by leveraging the reasoning and knowledge synthesis capabilities of a LLM to identify biologically relevant predictors, and the analytical power of a ML model (i.e., RF) to perform the quantitative prediction. In essence, SARAH uses AI twice: first to think, then to predict, illustrating how domain-specific intelligence can be operationalized into robust decision-support tools.

**Figure 1:**
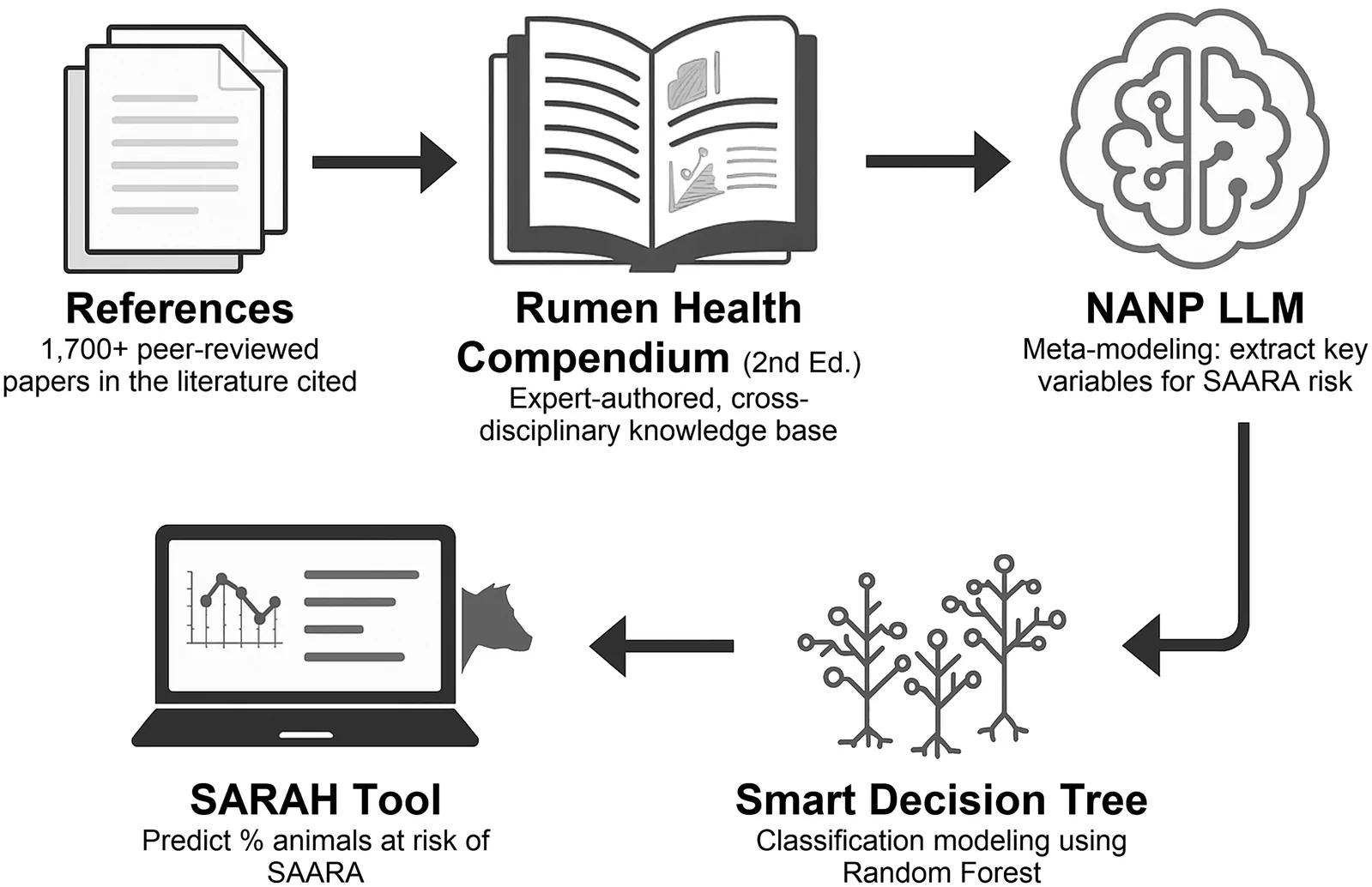
Workflow for developing the Smart Adviser for Rumen Acidosis and Health (SARAH). Scientific knowledge from peer-reviewed publications was consolidated into the Rumen Health Compendium book (Tedeschi and Nagaraja, 2025), which contained synthesized data, illustrations, and expert insights on rumen function and dysfunction. The Compendium and its cited literature were distilled into the NANP-LLM, a domain-specific large language model (LLM) developed by the National Animal Nutrition Program (NANP). In collaboration with field and academic experts, the NANP-LLM was used to identify 18 key animal, dietary, and environmental variables and their relationships, which informed the construction of classification models using random forest algorithms. These smart decision tree models were embedded into SARAH to predict the proportion of feedlot cattle at risk of subacute and acute ruminal acidosis (SAARA) (Tedeschi and Kaniyamattam 2025).

The smart decision support tool SARAH would allow users to run models with or without cross-validation, using datasets of varying sizes to simulate risk under different production conditions. Preliminary results show that while untrained RF models may display slightly higher raw accuracy, trained models provide greater stability, lower variability, and more reliable risk estimates, particularly when using at least 50,000 simulated records (Tedeschi and Kaniyamattam 2025). Beyond binary classification, SARAH incorporates concepts of area and time above and under the curve (ATAUC) to capture the dynamics of ruminal pH fluctuations, providing a biologically grounded estimate of the proportion of animals at risk within a feedlot pen (Tedeschi 2025b). By combining the biological depth of the peer-reviewed papers and the RHC insights, the structuring capabilities of the NANP-LLM, and robust ML methods, SARAH exemplifies how modern decision-support systems can bridge mechanistic knowledge with predictive analytics to guide proactive management of ruminal acidosis in cattle. Looking ahead, integrating SARAH’s data-driven architecture with mechanistic nutrition models could create a truly hybrid modeling framework (Tedeschi 2022, 2023), combining the interpretability and biological fidelity of process-based systems with the adaptive learning capacity of AI.

Together, these applications demonstrate that LLM are no longer peripheral in animal science research: they are increasingly being adapted for real-world problems in nutrition, health, breeding, and sustainability. However, challenges remain. Many tools are still experimental, domain-specific validation is sparse, and the pace of model innovation means current benchmarks can quickly become outdated. Achieving lasting impact will require greater integration with mechanistic models, rigorous bias testing, transparent documentation of training data, and continued domain-specific fine-tuning to ensure scientific reliability and relevance in animal agriculture.

## 4 Conclusions and implications

The integration of LLM into scientific workflows has already begun to reshape research methodologies, offering powerful tools for literature review automation, hypothesis generation, and data analysis. These advancements present significant opportunities for accelerating knowledge discovery and enhancing productivity across disciplines, including animal sciences, where LLM are being leveraged to refine nutrition models, optimize genetic selection, and improve disease surveillance. However, as LLM continue to evolve, their adoption must be approached with caution and responsibility, given the ethical concerns they raise—particularly in areas of bias, reproducibility, and the potential erosion of human expertise. In animal sciences, where research directly impacts food security, sustainability, and animal welfare, ensuring the reliability and accuracy of AI-generated insights is critical.

Ensuring scientific integrity in the era of AI-driven research requires a deliberate and balanced approach. While LLM can eenhance efficiency, they must complement human expertise rather than replace it. Transparency, accountability, and fairness in their deployment are paramount, particularly in maintaining the credibility of scientific discourse and ensuring that AI-assisted decision-making in livestock management and nutrition remains robust and evidence-based. Open-source LLM provide one possible solution to these concerns, offering greater reproducibility, accessibility, and validation opportunities. Moreover, proper attribution of AI-generated content, rigorous verification of outputs, and heightened awareness of biases must become standard practices to ensure that AI-generated knowledge remains trustworthy and ethically sound.

Moving forward, the scientific and agricultural community must continuously evaluate LLM capabilities and limitations, developing clear guidelines and best practices for their responsible use in animal science. Particularly promising will be the development of hybrid modeling frameworks that integrate mechanistic models with data-driven AI systems, linking biological interpretability with predictive power. By fostering open collaboration and ethical AI governance, researchers, industry professionals, and policymakers can harness the potential of LLM while upholding the core principles of scientific integrity, sustainability, and innovation in livestock production. Ultimately, most agricultural applications will not rely on full model training, but rather on more practical approaches, such as fine-tuning and augmentation.

## Abbreviations

AI: artificial intelligence
ATAUC: area and time above and under the curve
LLM: large language model
LSTM: long short-term memory
ML: machine learning
NANP: National Animal Nutrition Program
RAG: retrieval-augmented generation
RF: random forest
RHC: Rumen Health Compendium
RNN: recurrent neural networks
SAARA: subacute and acute ruminal acidosis
SARAH: Smart Adviser for Rumen Acidosis and Health
TMR: total mixed ration
